# Liver, visceral and subcutaneous fat in men and women of South Asian and white European descent: a systematic review and meta-analysis of new and published data

**DOI:** 10.1007/s00125-022-05803-5

**Published:** 2022-10-13

**Authors:** Stamatina Iliodromiti, James McLaren, Nazim Ghouri, Melissa R. Miller, Olof Dahlqvist Leinhard, Jennifer Linge, Stuart Ballantyne, Jonathan Platt, John Foster, Scott Hanvey, Unjali P. Gujral, Alka Kanaya, Naveed Sattar, Mary Ann Lumsden, Jason M. R. Gill

**Affiliations:** 1 Wolfson Institute of Population Health, Queen Mary University of London, London, UK; 2 School of Medicine, University of Glasgow, Glasgow, UK; 3 School of Cardiovascular and Metabolic Health, University of Glasgow, Glasgow, UK; 4 Worldwide Research Development and Medical, Pfizer, Cambridge, MA USA; 5 AMRA Medical AB, Linköping, Sweden; 6 Department of Medical and Health Sciences, Linköping University, Linköping, Sweden; 7 Department of Radiology, Greater Glasgow and Clyde NHS, Glasgow, UK; 8 Department of Clinical Physics and Bioengineering, Beatson West of Scotland Cancer Centre, Glasgow, UK; 9 Radiotherapy Physics, Derriford Hospital, Plymouth, UK; 10 Hubert Department of Global Health, Rollins School of Public Health, Emory University, Atlanta, GA USA; 11 Department of Medicine, University of California San Francisco, San Francisco, CA USA

**Keywords:** Abdominal, Computed tomography, Fat, Liver, Magnetic Resonance Imaging, Meta-analysis, South Asian, Systematic review, Visceral

## Abstract

**Aims/hypothesis:**

South Asians have a two- to fivefold higher risk of developing type 2 diabetes than those of white European descent. Greater central adiposity and storage of fat in deeper or ectopic depots are potential contributing mechanisms. We collated existing and new data on the amount of subcutaneous (SAT), visceral (VAT) and liver fat in adults of South Asian and white European descent to provide a robust assessment of potential ethnic differences in these factors.

**Methods:**

We performed a systematic review of the Embase and PubMed databases from inception to August 2021. Unpublished imaging data were also included. The weighted standardised mean difference (SMD) for each adiposity measure was estimated using random-effects models. The quality of the studies was assessed using the ROBINS-E tool for risk of bias and overall certainty of the evidence was assessed using the GRADE approach. The study was pre-registered with the OSF Registries (https://osf.io/w5bf9).

**Results:**

We summarised imaging data on SAT, VAT and liver fat from eight published and three previously unpublished datasets, including a total of 1156 South Asian and 2891 white European men, and 697 South Asian and 2271 white European women. Despite South Asian men having a mean BMI approximately 0.5–0.7 kg/m^2^ lower than white European men (depending on the comparison), nine studies showed 0.34 SMD (95% CI 0.12, 0.55; *I*^2^=83%) more SAT and seven studies showed 0.56 SMD (95% CI 0.14, 0.98; *I*^2^=93%) more liver fat, but nine studies had similar VAT (−0.03 SMD; 95% CI −0.24, 0.19; *I*^2^=85%) compared with their white European counterparts. South Asian women had an approximately 0.9 kg/m^2^ lower BMI but 0.31 SMD (95% CI 0.14, 0.48; *I*^2^=53%) more liver fat than their white European counterparts in five studies. Subcutaneous fat levels (0.03 SMD; 95% CI −0.17, 0.23; *I*^2^=72%) and VAT levels (0.04 SMD; 95% CI −0.16, 0.24; *I*^2^=71%) did not differ significantly between ethnic groups in eight studies of women.

**Conclusions/interpretation:**

South Asian men and women appear to store more ectopic fat in the liver compared with their white European counterparts with similar BMI levels. Given the emerging understanding of the importance of liver fat in diabetes pathogenesis, these findings help explain the greater diabetes risks in South Asians.

**Funding:**

There was no primary direct funding for undertaking the systematic review and meta-analysis.

**Graphical abstract:**

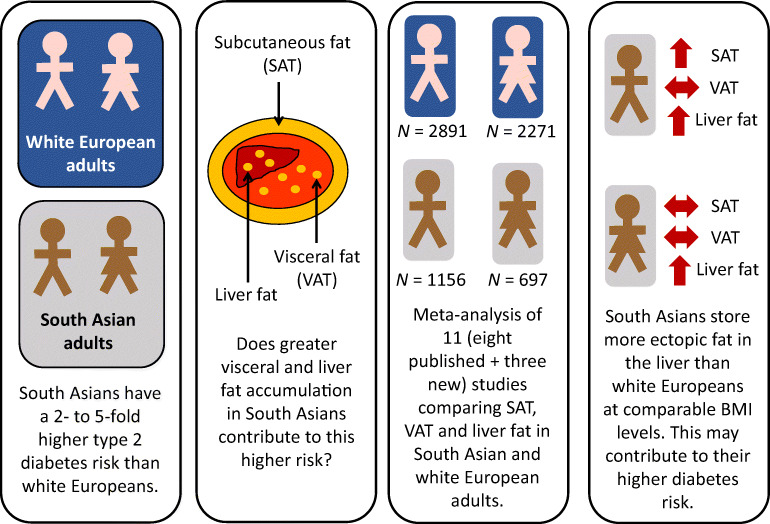

**Supplementary Information:**

The online version contains peer-reviewed but unedited supplementary material available at 10.1007/s00125-022-05803-5.

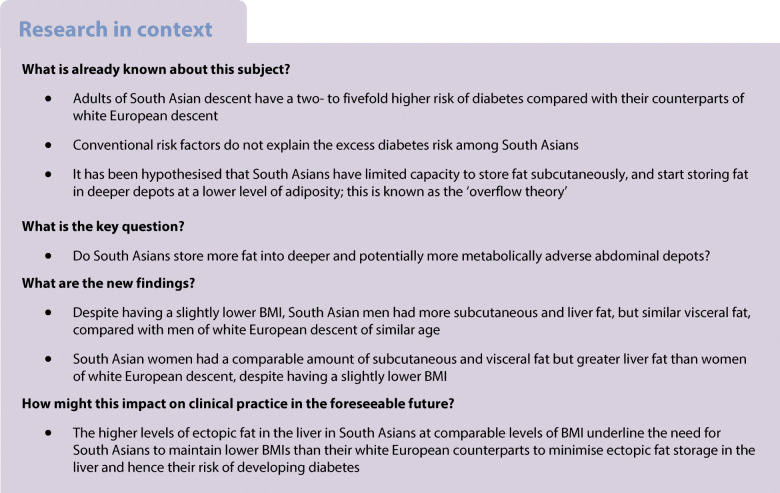



## Introduction

South Asians living in Europe and North America have a two- to fivefold higher risk of developing type 2 diabetes than their counterparts of white European descent living in the same countries and develop the disease at a younger age and lower BMI [1–3]. Furthermore, South Asians exhibit a 30–100% higher mortality risk for coronary heart disease and cardiovascular disease than their white European counterparts [4–6]. In addition, South Asians without diabetes have higher fasting glycaemic indices than white Europeans, and greater levels of insulin resistance [7, 8]. Conventional cardiometabolic factors do not account for the magnitude of the inter-ethnic differences in the burden of type 2 diabetes and cardiovascular disease. Smoking is less prevalent among South Asians [8], but overall caloric intake appears not to differ meaningfully between the two ethnic groups, with South Asians consuming larger quantities of polyunsaturated fats [9]. Diabetes rates are also increasing rapidly in all South Asian countries.

It has been suggested that increased central adiposity and storage of fat in deeper abdominal compartments, such as around the viscera or liver [1, 10], may be a key pathway leading to greater insulin resistance and subsequent type 2 diabetes and cardiovascular disease in South Asians. Some authors have hypothesised that South Asians have a lower capacity to store fat subcutaneously, leading to earlier ‘spill-over’ into harmful secondary visceral and ectopic depots, the so-called ‘adipose tissue overflow’ hypothesis [11, 12]. However, the evidence from studies comparing the fat distribution in the two ethnic groups is conflicting; one study suggests that South Asians store more fat subcutaneously [13], whilst another suggests that they accumulate excess fat both subcutaneously and intra-abdominally [14], and another showing no substantial difference in fat depots between the two groups [12]. The fact that many of those studies were relatively small and thus lack of power, together with differences in study characteristics, may have contributed to the discrepancy in the findings. The aim of our study was to systematically collate all existing published data comparing the amounts of subcutaneous (SAT) and visceral (VAT) adipose tissue and liver fat between South Asian and white European adults, and supplement this with unpublished data from our group and the UK Biobank study, to provide the most robust assessment to date of potential ethnic differences in the levels of fat in key metabolic fat compartments.

## Methods

The study, which was pre-registered with the OSF Registries (https://osf.io/w5bf9), was conducted according to the PRISMA guidelines [15], and followed a structured protocol that was agreed among the authors in advance of the literature search. Data eligible for meta-analysis included both original research and existing publications identified by systematic review.

### Original research

Unpublished data from two studies undertaken by the authors were included in the meta-analysis. Both studies were cross-sectional and assessed the lifestyle and cardiometabolic risk factors of South Asian and white European men and women, without diabetes, aged 40–70 years, who lived in Scotland (UK). Both studies have been described in detail elsewhere [8, 16], and involved radiological assessment of fat distribution in men and women. The methodology for fat measurement and the demographic characteristics of participants with radiological assessment are shown in electronic supplementary material (ESM) Methods and ESM Table 1). In addition, we included new data from the UK Biobank. UK Biobank is a large prospective study that recruited 502,643 participants (response rate 5.5%) between 2006 and 2010, age range 37–73 years, and consented for their records to be linked with routine data (hospital admissions and death registries). Participants attended one of 22 assessment centres across the UK, where they completed a touch screen questionnaire, had physical measurements taken, and provided biological samples as described in detail elsewhere [17, 18]. The UK Biobank imaging study began in 2014, and intends to collect imaging data of the vital organs, including MRI measures of abdominal body fat, by recalling 100,000 participants. At the time of performing the analyses for this study, abdominal MRI data were available for approximately 30,000 participants. We used abdominal imaging data from South Asians without diabetes who were matched for age, sex and BMI with white Europeans without diabetes in a 1:5 ratio to maximise statistical power. The protocol for abdominal fat measurement in the UK Biobank imaging study has been published elsewhere [19, 20].

### Systematic review of published data and selection criteria

To identify existing publications, we searched the Embase and PubMed databases from inception to August 2021, combining the MeSH terms ‘obesity’, ‘adipocyte’, ‘liver’, ‘south asia’, ‘asian continental ancestry group’, ‘caucasian’ and ‘european’, and using the keywords ‘obes*’, ‘fat*’, adipos*’, ‘liver?fat*’, ‘fatty?liver*’, ‘south?asia*’, ‘india*’, ‘bangladesh*’, ‘sri?lanka*’, ‘pakistan*’, ‘caucasian*’, ‘white*’ and ‘european*’ with Boolean rules. A search filter for studies related to humans with a restriction to English language was included. Two researchers (JM and SI) screened all the titles and abstracts, and studies were read in full when they fulfilled the selection criteria. The reference lists of eligible studies were hand-searched to find further relevant studies. Grey literature was also searched via the OpenGrey website (https://opengrey.eu/).

We included studies that met the following criteria: (1) participants were men or women aged over 18 years; (2) participants had measurements of abdominal SAT and VAT, and/or liver fat by computed tomography (CT) or MRI; (3) the study included a South Asian group and a comparison group of white European descent; and (4) any study design apart from case reports. South Asian ethnic background was either reported as such in the studies or participants were of Indian, Pakistani, Bangladeshi or Sri Lankan background. In the meta-analysis, we included studies for which we could extract mean values and standard deviations from published or requested data. We only included data stratified by sex. Two researchers (JM and SI) independently assessed the papers for final selection. Any discrepancies were resolved by discussion. A third reviewer (JMRG) was consulted if any unresolved issues persisted.

### Data extraction and quality assessment

We developed a data extraction spreadsheet that included the following information: study characteristics (first author, year of publication, number of people of South Asian descent and number of people of white European descent, study design), study sample characteristics (sex, mean age and BMI, mean fasting glucose and insulin, diagnosis of diabetes [yes or no]), test characteristics (method of measuring abdominal and/or liver fat, mean value for fat quantity and standardised mean difference [SMD] for each group). If the numerical data were not extractable from the published data, the authors were contacted via email. We were unable to obtain data for insulin and glucose concentrations for four studies [19, 21–25]. References [22–24] are multiple papers referring to one study dataset.

We used a preliminary version of the ROBINS-E tool (risk of bias in non-randomised studies of exposures) to assess the risk of bias in the individual studies selected across seven domains; the results for the individual studies were then summarised to provide an overall study-level assessment regarding the risk of bias (low, moderate, serious or critical) [26]. We also used the GRADE (Grading of Recommendations, Assessment, Development and Evaluations) approach to assess the overall certainty of evidence of the meta-analysis findings to provide an evidence certainty score (very low, low, moderate or high) [27].

### Data analysis

We used Stata software version 14.1 (Stata, USA) for statistical analysis. The weighted SMD (with 95% CI) was calculated by combining the mean differences in fat between the two groups in each study using a random-effects model. One study reported hepatic attenuation to assess liver fat, rather than the liver fat percentage [28]. As lower hepatic attenuation implies higher liver fat, the sign of the standardised mean ethnic difference in hepatic attenuation was reversed to make the findings comparable with other studies. Analyses were stratified by sex. We performed two sensitivity analyses: (1) by separating the studies that included any participants with diabetes from those without diabetes to assess whether the presence of diabetes modified the results, and (2) by only including the studies with matched BMI between the ethnic groups. We also performed an analysis stratified by assessment tool (CT vs MRI). Heterogeneity resulting from the mean difference in each study not being identical with the pooled estimate was quantified using the *I*^2^ measure [29].

We assessed the risk of publication bias and potential small-study effect by constructing funnel plots, which plot the mean difference from each study against the SEM as a measure of study size [30].

### Ethics

Previously unpublished data from studies by Iliodromiti et al and Ghouri et al were included in these analyses [8, 16]. Both studies were approved by the West of Scotland Research Ethics Committee, and performed according to the Declaration of Helsinki. All participants gave written informed consent to participate. The UK Biobank study was approved by the North West Multi-Centre Research Ethics Committee, and all participants provided written informed consent to participate. Ethical approval was not required for the analysis of data from previously published studies.

## Results

### Original research

Two of the studies included were studies performed by our group for which data on radiologically assessed adiposity measures had not previously been published. The methodology of fat measurement for these two studies is described in ESM Methods. ESM Table 1 summarises the demographic and cardiometabolic profile of the participants with radiological data from the unpublished studies by Ghouri et al and Iliodromiti et al. Other data from these studies have been reported previously [8, 16].

### Systematic search results

**Fig. 1:**
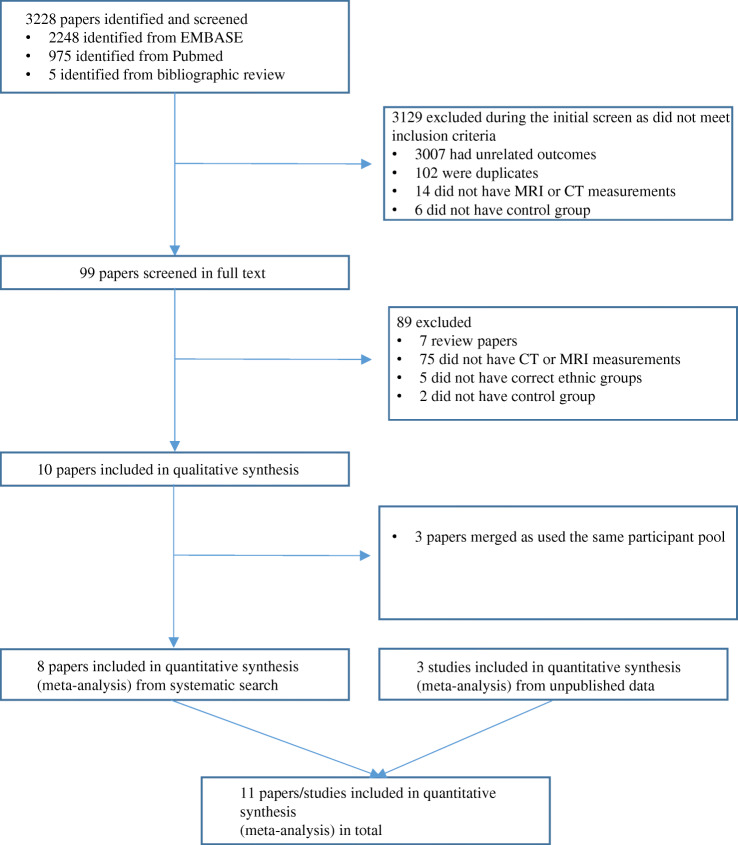
Flow chart of the search strategy

Figure 1 shows the search and numerical selection flowchart. The systematic search of the biomedical databases resulted in 3228 hits; including 2248 from the Embase search and 975 from PubMed. Five additional studies were identified by bibliographic search. Of these, 99 papers were selected and read in full, of which 89 were excluded for a variety of reasons as detailed in Fig. 1. Therefore, 11 studies (with one study contributing two different but not overlapping datasets [21]) including data from the UK Biobank, were finally selected for the meta-analyses (*n*=4047 men and 2968 women for SAT and VAT comparisons and *n*=3071 men and 2651 women for liver fat comparison) [12, 13, 21–25, 31, 32]. The papers by Kohli and Lear and Dick et al [23, 24] refer to the same study, data for which were initially published by Lear et al [22]. The study by Shah et al [28] did not present data stratified by sex, but the authors kindly shared stratified results after we contacted them by email.

### Description of studies

Table 1 summarises the characteristics of the studies included in the systematic review. ESM Tables 2 and 3 summarise the mean age, BMI and fasting glucose and insulin levels (when available) for all the included studies, stratified by sex and ethnicity. The mean age did not differ between ethnic groups of either sex. South Asian men had a mean BMI that was approximately 0.7 kg/m^2^ lower for the SAT and VAT comparisons and approximately 0.5 kg/m^2^ lower for the liver fat comparison compared with their white European counterparts. South Asian women had a mean BMI that was approximately 0.9 kg/m^2^ lower for SAT, VAT and liver fat comparisons compared with their white European counterparts.

**Table 1:** Characteristics of the included studies

Reference	AFM mode	AFM location	LFM	Sex	Sample size (*n*)	Age range (years)	BMI (kg/m^2^)	Glucose and insulin concentrations	Diabetes?
Anand et al (2011) [12]	MRI (1.5 T Symphony, Siemens)	Mid-L4	Proton MRS	Men and women	Men: SA 32, E 21 Women: SA 24, E 31	31–40	Men: SA 27.2 ± 0.7 E 27.7 ± 0.9 Women: SA 25.7 ± 1.2 E 29.1 ± 1.0	Fasting glucose (mmol/l) Men: SA 5.1 ± 0.5 E 5.0 ± 0.5 Women: SA 4.9 ± 0.5 E 4.9 ± 0.5 Log_e_ Fasting insulin (pmol/l) Men: SA 4.2 ± 0.6 E 3.8 ± 0.6 Men: SA 4.0 ± 0.9 E 4.0 ± 0.8	No
Bakker et al (2014) [32]^a^	MRI (1.5 T Gyroscan ACS-NT15, Philips)	L5	No	Men	SA 12, E 12	19–25	SA = 22.2 ± 0.6 E = 20.9 ± 0.6	Fasting glucose (mmol/l) SA 5.2 ± 0.3 E 5.1 ± 0.3 *Fasting insulin* (pmol/l) SA 49 (29) E 34 (32)	No
Chandalia et al (2007) [13]	MRI (1.5 T Gyroscan Intera, Philips)	Total abdominal volume	No	Men	SA 29, E 18	21–30	SA 24 ± 4 E 23 ± 3	Fasting glucose (mmol/l) SA 5.3 ± 0.3 E 5.0 ± 0.3	No
Eastwood et al (2013) [21]	CT (MX 8000 IDT64 detector, Philips)	Mid-L4	No	Men and women	Men: SA 439, E 517 Women: SA 75, E 152	61–76	Men: SA 26 ± 4 E 28 ± 4 Women: SA 28 ± 5 E 28 ± 5	No	Yes
Lear et al (2007) [22], Kohli and Lear (2013) [23], Dick et al (2013) [24]	CT (CTi Advantage scanner, General Electric)	L4–L5	CT (CTi Advantage scanner, General Electric)	Men and women	SAT/VAT Men: SA 102, E 100 Women: SA 97, E 102 Liver Men: SA 66, E 66 Women: SA 66, E 75	30–65	Men: SA 27.9 ± 4.5 E 27.7 ± 4.6 Women: SA 27.7 ± 5.3 E 27.7 ± 5.5	No	No
Petersen et al (2006) [25]	NA	NA	Proton MRS (2.1 or 4 T Biospec, Bruker Instruments)	Men	SA 23, E 73	20–30	SA 22.8 ± 2.2 E 22.5 ± 2.2	No	No
Shah et al (2016) [28]^b^	CT (Philips, Toshiba or Siemens, depending on site)	Between L4 and L5	CT (Philips, Toshiba or Siemens, depending on site)	Men and women	Men: SA 309, E 1164 Women: SA 318, E 1300	40–84	Men: SA 25.8 ± 4.1 E 27.7 ± 3.9 Women: SA 25.8 ± 4.6 E 27.3 ± 5.7	Fasting glucose (men) (mmol/l) SA 5.4 ± 0.6 E 5.0 ± 0.6 Fasting glucose (women) (mmol/l) SA 5.2 ± 0.5 E 4.8 ± 0.6 Fasting insulin (men) (pmol/l) SA 62 ± 40 E 57 ± 68 Fasting insulin (women) (pmol/l) SA 54 ± 41 E 52 ± 69	No
Szuszkiewicz-Garcia et al (2012) [31]	MRI (1.5 T Gyroscan Intera, Philips)	Total abdominal volume	No	Women	SA 16, E 16	21–30	SA 22 ± 4 E 23 ± 5	Fasting glucose (mmol/l) SA 4.8 ± 0.4 E 4.8 ± 0.4 Fasting insulin (pmol/l) SA 76 ± 34 E 83 ± 48	No
UK Biobank (unpublished data) [19] ^c^	MRI (1.5 T Siemens)	Total abdominal	Proton MRS (1.5 T Siemens)	Men and women	Men: SA 207, E 1035 Women: SA 123, E 615	39–70	Men: SA 25.6 ± 3.4 E 25.6 ± 3.4 Women: SA 25.5 ± 4.1 E 25.5 ± 4.0	No	No
Ghouri et al (unpublished data)	MRI (3 T Magnetom, Siemens)	L3–L5	Proton MRS (3 T Magnetom, Siemens)	Men	SAT/VAT SA 26, E 24 Liver SA 28, E 23	40–60	SA 28.5 ± 4.8 E 27.4 ± 3.2	Fasting glucose (mmol/l) SA 5.25 (4.90–5.70) E 5.00 (4.80–5.55) Fasting insulin (pmol/l) SA 91 (58–118) E 61 (40–74)	No
Iliodromiti et al (unpublished data)	MRI (3 T Magnetom, Siemens)	L3–L5	Proton MRS (3 T Magnetom, Siemens)	Women	SA 44, E 55	40–70	SA 27 ± 5.3 E 27.1 ± 4.5	Fasting glucose (mmol/l) SA 5.00 (4.60–5.30) E 5.00 (4.70–5.20) Fasting insulin (pmol/l) SA 60 (41–88) E 44 (31–58)	No

Data for BMI, glucose and insulin are means ± SD or median (IQR)

All studies were cross-sectional

^a^A cross-sectional analysis of baseline data from a non-randomised controlled trial

^b^A cross-sectional analysis of two cohort studies, MESA (Multi-Ethnic Study of Atherosclerosis) and MASALA (Mediators of Atherosclerosis in South Asians Living in America)

^c^A cross-sectional analysis of baseline data from a cohort study, imaging subset of total UK Biobank participants

AFM, abdominal fat measurement; E, European; L, lumbar; LFM, liver fat measurement; MRS, magnetic resonance spectroscopy; SA, South Asian

### Quality assessment

ESM Tables 4 and 5 present the study-level judgements of bias using the ROBINS-E tool for the SAT and VAT, and the liver fat outcomes, respectively. Four studies for SAT and VAT and two studies for liver fat outcomes were rated at moderate risk of confounding due to differences in BMI between ethnic groups for one or both sexes. In all instances where this occurred, the BMI values were lower in the South Asian group, which would have acted to bias the differences between the ethnic groups in the outcome towards the null. One study was rated as being at serious risk of confounding due to inclusion of participants with diabetes in the sample and BMI differences between groups. All studies, except UK Biobank in which outcome measures of SAT, VAT and liver fat were obtained using an automated algorithm, were rated as having a moderate risk of bias for the measurement of outcomes, as these measures were not reported to have been undertaken in a blinded manner, which may have biased findings against the null hypothesis as assessors may have expected more ectopic fat in South Asian participants. Thus, the overall study-level bias was rated as moderate for all studies, except that by Eastwood et al [21], which was rated as having serious risk of bias, and the UK Biobank study, which was rated as having low risk of bias. ESM Table 6 summarises the certainty of evidence for studies included in meta-analysis as assessed using the GRADE approach. The overall certainty of evidence from summary findings of the meta-analysis was assessed as moderate due to heterogeneity, study limitations/bias, and possible publication bias for the SAT/VAT outcomes (see below). However, in the sensitivity analyses described below, exclusion of studies that included participants with diabetes, and only including studies in which BMI was matched between ethnic groups, did not materially affect the findings. Factors that increased the summary certainty of evidence from low to moderate included large numbers of participants, the size of effect, precision and directness.

### Meta-analysis

We summarised imaging data on SAT and VAT from 1156 South Asian men and 2891 white European men (of comparable age but the mean BMI in South Asians was approximately 0.7 kg/m^2^ lower). We also compared data on liver fat from 677 South Asian men vs 2394 white European men (of comparable age but the mean BMI in South Asians was approximately 0.5 kg/m^2^ lower). For women, we compared the data on SAT and VAT from 697 South Asian participants vs 2271 white European participants (of comparable age but the mean BMI in South Asians was approximately 0.9 kg/m^2^ lower), and data on for liver fat from 575 South Asian participants vs 2076 white European participants (of comparable age but the mean BMI in South Asians was approximately 0.9 kg/m^2^ lower).

**Fig. 2:**
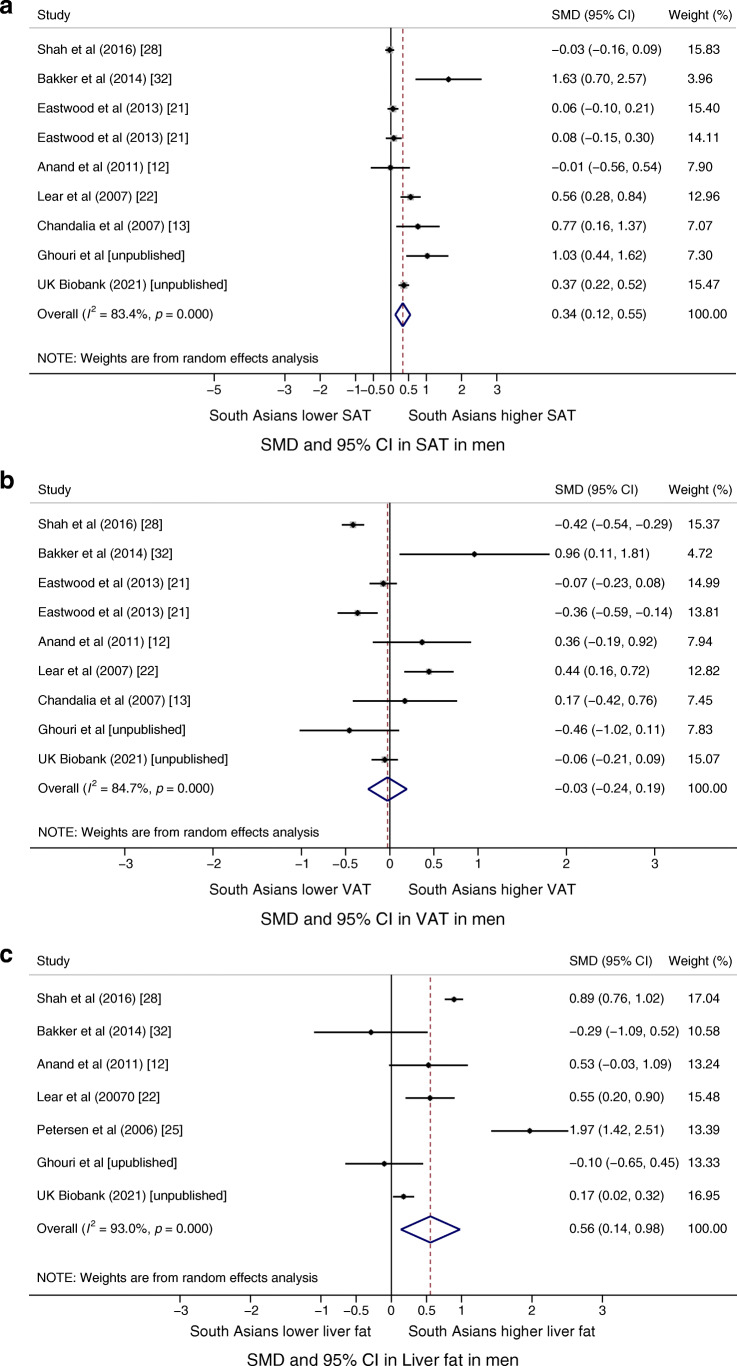
SMDs in (**a**) SAT, (**b**) VAT and (**c**) liver fat in South Asian vs white European men

**Fig. 3:**
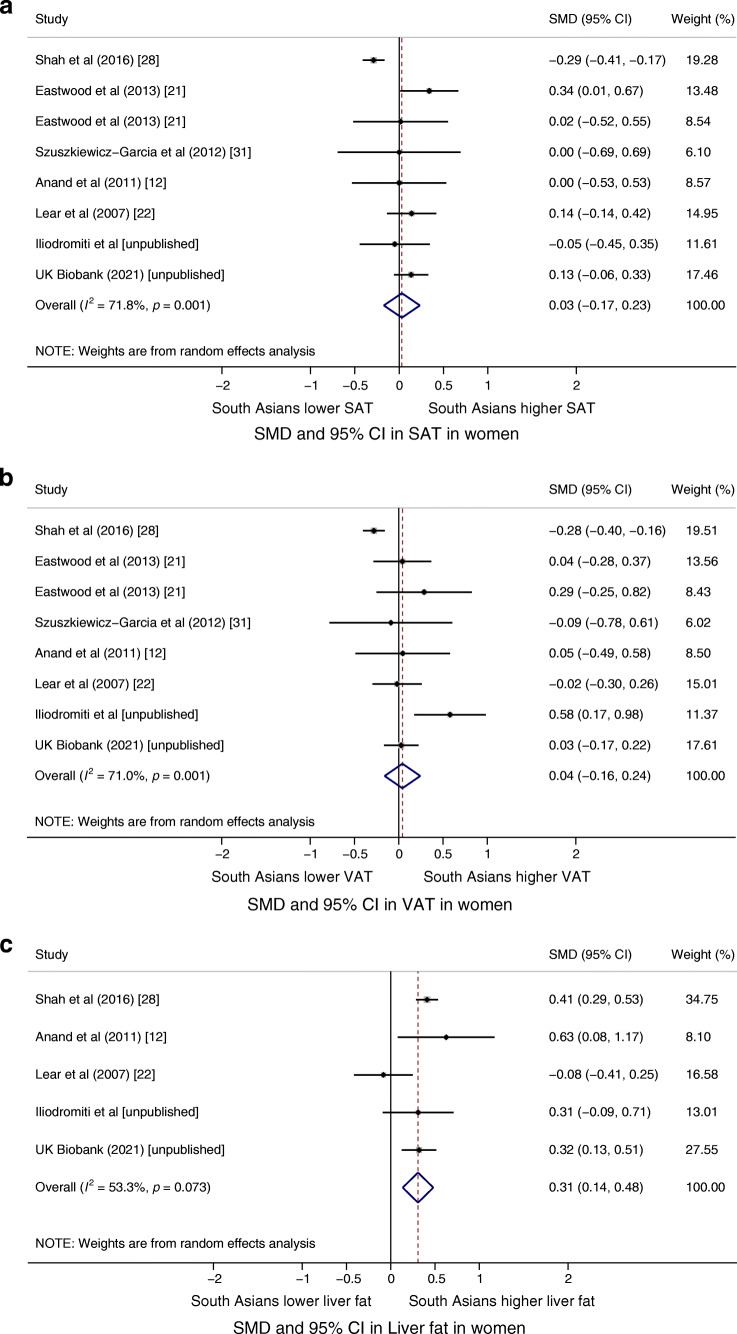
SMDs in (**a**) SAT, (**b**) VAT and (**c**) liver fat in South Asian vs white European women

Figure 2 shows the SMD in fat in men. In nine studies, South Asian men had 0.34 SMD (95% CI 0.12, 0.55; *I*^2^=83%; *p*<0.001) more SAT than their white European counterparts. In seven studies, South Asian men had 0.56 SMD (95% CI 0.14, 0.98; *I*^2^=93%; *p*<0.001) more liver fat than their white European counterparts. There was no substantial difference in VAT between South Asian and white European participants in nine studies (SMD −0.03; 95% CI −0.24, 0.19; *I*^2^=85%; *p*<0.001). All meta-analyses in men showed high heterogeneity. Figure 3 shows the SMD in fat in women. There was no substantial difference between South Asian and white European participants in eight studies of SAT or VAT (SMD 0.03; 95% CI −0.17, 0.23; *I*^2^=72%; *p*=0.001 and SMD 0.04; 95% CI −0.16, 0.24; *I*^2^=71%; *p*=0.001, respectively). In five studies, South Asian women had 0.31 SMD (95% CI 0.14, 0.48; *I*^2^=53%; *p*=0.07) more liver fat than their white European counterparts. For women, all meta-analyses showed high heterogeneity, except for the liver fat data, which showed moderate heterogeneity.

#### Sensitivity analysis

No studies investigating liver fat included any participants with diabetes. When we compared data for VAT and SAT in South Asian vs white European men and women after excluding data from the one study that included participants with diabetes [21], the results did not materially change for either sex (ESM Figs 1 and 2). For the studies with matched BMI between the two ethnic groups, point estimates for the standardised differences in SAT and liver fat between South Asian and white European men were similar to those observed in analyses including all studies (ESM Figs 3 and 4), although the 95% CI were wider. Findings were similar in studies using MRI vs CT as the assessment tool (ESM Figs 5–8).

#### Publication bias

ESM Fig. 9 presents funnel plots for each main analysis, suggesting symmetry and therefore a small likelihood of publication bias or small-study effect for VAT and liver fat for men and liver fat for women. We cannot exclude the possibility of publication bias or a small-study effect for SAT and VAT for women and SAT for men, with the asymmetry in the funnel plots suggesting that small studies showing greater abdominal fat for white European participants may be lacking.

## Discussion

To our knowledge, this evidence synthesis, including data from 1853 participants of South Asian descent and 5162 participants of white European descent, is the largest analysis comparing robust imaging data (CT or MRI) of various abdominal fat compartments between South Asian and white European adults. These data suggest that both South Asian men and women store greater ectopic fat in the liver at a lower BMI compared with their counterparts of white European descent, and that there may be a sex-specific difference in ethnic distribution of SAT. South Asian men had greater amounts of SAT and ectopic fat accumulated in the liver than their white European counterparts despite having a slightly lower BMI, although this was not clearly accompanied by higher levels of VAT. In women, there was no substantial difference in SAT or VAT distribution between South Asians and white European participants; however, like men, South Asian women had more ectopic fat in the liver compared with their white European counterparts, despite having a BMI that was approximately 0.9 kg/m^2^ lower. The slightly lower BMI in the South Asian participants compared with white European participants in these studies may have contributed to the absence of a difference in VAT between the two ethnic groups. In the subset of studies where the BMI did not differ between the ethnic groups [16, 19, 22–25], South Asian men and women showed a numerically higher level of VAT, as well as higher levels of SAT and ectopic liver fat, compared with men and women of white European descent, but the statistical power in these subgroup analyses was limited. Thus, taking all data together, we can be most confident about the finding of higher liver fat levels in South Asian participants, as there were similar findings in both South Asian men and women relative to their white European counterparts, and broadly concordant findings in the subgroups of those without diabetes or matched for BMI. In addition, the liver analyses showed a low likelihood of publication bias or small-study effect. However, given the available data, our conclusions about ethnic differences in VAT are more cautious.

The central role of the liver in diabetes pathogenesis has become increasingly apparent in recent years, with the organ being a site of excess fat storage in those with hyperinsulinaemia due to either genetic or familial factors, with consequent excessive hepatic gluconeogenesis [33]. It has been shown that surrogate markers of liver fat and their change over time predict diabetes [33, 34], whereas substantial weight loss from use of low-energy diets can lead to rapid fat loss from the liver and improved insulin sensitivity in people with diabetes [34]. These studies were performed predominantly in participants of white European origin, and align with the importance of liver fat in the pathogenesis of diabetes in this ethnic group, as well with molecular mechanisms whereby fat-derived metabolites impair insulin signalling [35]. Export of excessive triacylglycerol from the liver may also be a key feature in the beta cell dysfunction in those who develop diabetes [33], and South Asians are known to have elevated circulating triacylglycerol levels at similar levels of BMI compared with white Europeans [36]. More recently, genetic studies have further suggested a causal role for liver fat in the pathogenesis of type 2 diabetes [37].

Greater SAT at a lower BMI in South Asian men implies there must be lower lean muscle mass in this group, which is an additional independent risk factor for type 2 diabetes [38], and other data has shown that lower lean mass contributes to the higher levels of insulin resistance observed in South Asians compared with other ethnic groups [39]. Clearly, in view of the present findings, more work on understanding ethnic differences in ectopic fat is urgently needed, including examining why South Asians appear to accumulate liver fat more rapidly at lower BMIs, and whether excess liver fat can be reversed by lifestyle measures, in particular intentional weight loss, in this group.

According to the ‘adipose tissue overflow’ hypothesis [11, 12], fat deposition starts predominantly in the subcutaneous region until inflammatory mediators halt the recruitment of new adipocytes. At this point, the capacity of subcutaneous tissue for further fat storage is reduced, and positive energy balance leads to an overflow of fatty acids to deeper adipose compartments (i.e. visceral) or ectopic tissues (i.e. hepatic). The ‘tipping’ point at which subcutaneous tissue reaches its maximum storage capacity is thought to vary for each individual, and depends on genetic and environmental factors [40], and it has been hypothesised that this occurs at a lower BMI in South Asians [11, 12]. The present findings are partially in agreement with this. South Asian participants of both sexes accumulated more ectopic fat in the liver at similar or lower BMI than white European participants. However, South Asian men also had higher levels of SAT, so the relative importance of a lower capacity for SAT storage vs greater overall adipose tissue accumulation at a given BMI in terms of higher liver fat levels is unclear. Nevertheless, data suggest that South Asian men have larger adipocytes in their subcutaneous compartment compared with their white European counterparts even when they are matched for total and abdominal body fat [13]. Thus, it is plausible and consistent with our findings that the subcutaneous adipocytes in South Asian men have the capacity to become more hypertrophic and therefore allow accumulation of more fat in superficial depots. In addition, hypertrophic adipocytes are associated with greater insulin resistance, which may be the mediating pathway in the development of type 2 diabetes [13].

### Strengths and weaknesses

To our knowledge, this is the first study pooling imaging data from abdominal fat compartments in a large group of South Asian participants and comparing this with data from individuals of white European origin. We only included data obtained using CT and MRI, which are considered the gold standards for measuring abdominal fat, to minimise heterogeneity and measurement bias. We used an extensive search to ensure all the available relevant published and unpublished studies were included. However, we used a filter to restrict searches to ‘humans’ and ‘English language’. While it is unlikely that studies including both South Asians and a white European comparator group would not be published in English, the use of filters may have excluded very recently completed studies that had not yet completed the MEDLINE indexing process. Although the process of systematic review and meta-analysis is a robust way of estimating the true difference with less random error because of increased sample size, the mean differences estimated by the pooled data are subject to the limitations of the primary studies. Between-study heterogeneity may be self-limiting when pooling studies together to estimate a summary measure; however, we calculated the pooled estimate by using a random-effects model that accounts for unexplained heterogeneity within studies. We used established methodology to assess the impact of small-study bias on our pooled estimates and acknowledge that some potential biases may have occurred, although liver estimates, the most interesting and novel finding in our study, appear not to be meaningfully influenced. In addition, the results were similar in men and women, lending confidence that the findings are real. The sensitivity analysis on a subset of studies that included participants matched for BMI had limited power but showed biologically plausible results that South Asians of both sexes store more fat in all fat depots for any given BMI compared with their white European counterparts. The same was true when we examined data in those without diabetes.

### Conclusion

We conclude that both South Asian men and women store more fat in ectopic depots (liver) at a lower or comparable BMI than their counterparts of white European origin. South Asian men, but not women, appear to accumulate more fat superficially compared with their white European counterparts, but evidence for ethnic differences in VAT accumulation was less clear-cut, with no statistically significant differences between ethnic groups observed for this outcome.

Given our knowledge of the importance of liver fat in diabetes, the excess liver fat at a lower BMI in the South Asians compared with their counterparts of white European descent may be a key factor contributing to the development of insulin resistance and type 2 diabetes at lower levels of overall adiposity in South Asians. Further work is now needed to understand why South Asians accumulate liver fat more readily and at lower BMIs than their counterparts of white European descent, and to what extent weight loss interventions can normalise liver fat and blood glucose levels as they have been shown to do in white Europeans.

## Supplementary information

ESM (PDF 1350 kb)

## Data Availability

The datasets generated and analysed during the current study are available from the corresponding author on reasonable request.

## Abbreviations

CT: Computed tomography
SAT: Subcutaneous adipose tissue
SMD: Standardised mean difference
VAT: Visceral adipose tissue
GRADE: Grading of Recommendations, Assessment, Development and Evaluations approach

## References

[1] McKeigue PM, Shah B, Marmot MG (1991). Relation of central obesity and insulin resistance with high diabetes prevalence and cardiovascular risk in South Asians. Lancet.

[2] Sproston K, Mindell J (2006) The health of minority ethnic groups. In: Health Survey for England 2004 Vol Volume 1. National centre for social research Leeds, UK, DOI: 10.1128/AEM.72.1.144-149.2006

[3] Misra R, Patel T, Kotha P (2010). Prevalence of diabetes, metabolic syndrome, and cardiovascular risk factors in US Asian Indians: results from a national study. J Diabetes Complicat.

[4] Wild S, McKeigue P (1997). Cross sectional analysis of mortality by country of birth in England and Wales, 1970-92. BMJ (Clin Res ed).

[5] McKeigue PM, Miller GJ, Marmot MG (1989). Coronary heart disease in south Asians overseas: a review. J Clin Epidemiol.

[6] Wild SH, Fischbacher C, Brock A, Griffiths C, Bhopal R (2007). Mortality from all causes and circulatory disease by country of birth in England and Wales 2001-2003. J Public Health (Oxford, England).

[7] Gray LJ, Yates T, Davies MJ (2011). Defining obesity cut-off points for migrant South Asians. PLoS One.

[8] Ghouri N, Purves D, McConnachie A, Wilson J, Gill JM, Sattar N (2013). Lower cardiorespiratory fitness contributes to increased insulin resistance and fasting glycaemia in middle-aged South Asian compared with European men living in the UK. Diabetologia.

[9] McKeigue PM, Marmot MG, Adelstein AM (1985). Diet and risk factors for coronary heart disease in Asians in northwest London. Lancet.

[10] Sattar N, Gill JM (2015). Type 2 diabetes in migrant south Asians: mechanisms, mitigation, and management. Lancet Diabetes Endocrinol.

[11] Sniderman AD, Bhopal R, Prabhakaran D, Sarrafzadegan N, Tchernof A (2007). Why might South Asians be so susceptible to central obesity and its atherogenic consequences? The adipose tissue overflow hypothesis. Int J Epidemiol.

[12] Anand SS, Tarnopolsky MA, Rashid S (2011). Adipocyte hypertrophy, fatty liver and metabolic risk factors in South Asians: the Molecular Study of Health and Risk in Ethnic Groups (mol-SHARE). PLoS One.

[13] Chandalia M, Lin P, Seenivasan T (2007). Insulin resistance and body fat distribution in South Asian men compared to Caucasian men. PLoS One.

[14] Kohli S, Sniderman AD, Tchernof A, Lear SA (2010). Ethnic-specific differences in abdominal subcutaneous adipose tissue compartments. Obesity (Silver Spring, Md).

[15] Liberati A, Altman DG, Tetzlaff J (2009). The PRISMA statement for reporting systematic reviews and meta-analyses of studies that evaluate healthcare interventions: explanation and elaboration. BMJ (Clin Res ed).

[16] Iliodromiti S, Ghouri N, Celis-Morales CA, Sattar N, Lumsden MA, Gill JM (2016). Should physical activity recommendations for South Asian adults be ethnicity-specific? Evidence from a cross-sectional study of South Asian and White European men and women. PLoS One.

[17] Sudlow C, Gallacher J, Allen N (2015). UK biobank: an open access resource for identifying the causes of a wide range of complex diseases of middle and old age. PLoS Med.

[18] Palmer LJ (2007). UK Biobank: bank on it. Lancet.

[19] Linge J, Borga M, West J (2018). Body composition profiling in the UK biobank imaging study. Obesity (Silver Spring, Md).

[20] West J, Dahlqvist Leinhard O, Romu T (2016). Feasibility of MR-based body composition analysis in large scale population studies. PLoS One.

[21] Eastwood SV, Tillin T, Wright A (2013). Estimation of CT-derived abdominal visceral and subcutaneous adipose tissue depots from anthropometry in Europeans, South Asians and African Caribbeans. PLoS One.

[22] Lear SA, Humphries KH, Kohli S, Chockalingam A, Frohlich JJ, Birmingham CL (2007). Visceral adipose tissue accumulation differs according to ethnic background: results of the Multicultural Community Health Assessment Trial (M-CHAT). Am J Clin Nutr.

[23] Kohli S, Lear SA (2013). Differences in subcutaneous abdominal adiposity regions in four ethnic groups. Obesity (Silver Spring, Md).

[24] Dick TJ, Lesser IA, Leipsic JA, Mancini GB, Lear SA (2013). The effect of obesity on the association between liver fat and carotid atherosclerosis in a multi-ethnic cohort. Atherosclerosis.

[25] Petersen KF, Dufour S, Feng J (2006). Increased prevalence of insulin resistance and nonalcoholic fatty liver disease in Asian-Indian men. Proc Natl Acad Sci U S A.

[26] Schunemann HJ, Cuello C, Akl EA (2019). GRADE guidelines: 18. How ROBINS-I and other tools to assess risk of bias in nonrandomized studies should be used to rate the certainty of a body of evidence. J Clin Epidemiol.

[27] Guyatt GH, Oxman AD, Vist GE (2008). GRADE: an emerging consensus on rating quality of evidence and strength of recommendations. BMJ (Clin Res ed).

[28] Shah AD, Kandula NR, Lin F (2016). Less favorable body composition and adipokines in South Asians compared with other US ethnic groups: results from the MASALA and MESA studies. Int J Obes (2005).

[29] Higgins JP, Thompson SG, Deeks JJ, Altman DG (2003). Measuring inconsistency in meta-analyses. BMJ (Clin Res ed).

[30] Sterne JA, Sutton AJ, Ioannidis JP (2011). Recommendations for examining and interpreting funnel plot asymmetry in meta-analyses of randomised controlled trials. BMJ (Clin Res ed).

[31] Szuszkiewicz-Garcia M, Li R, Grundy SM, Abate N, Chandalia M (2012). Fat distribution and insulin resistance in young adult nonobese Asian Indian women. Metab Syndr Relat Disord.

[32] Bakker LE, van Schinkel LD, Guigas B (2014). A 5-day high-fat, high-calorie diet impairs insulin sensitivity in healthy, young South Asian men but not in Caucasian men. Diabetes.

[33] Taylor R, Al-Mrabeh A, Sattar N (2019) Understanding the mechanisms of reversal of type 2 diabetes. Lancet Diabetes Endocrinol. 10.1016/s2213-8587(19)30076-2 10.1016/S2213-8587(19)30076-2 31097391

[34] Taylor R, Al-Mrabeh A, Zhyzhneuskaya S (2018). Remission of human type 2 diabetes requires decrease in liver and pancreas fat content but is dependent upon capacity for beta cell recovery. Cell Metab.

[35] Smith U (2002). Impaired (‘diabetic’) insulin signaling and action occur in fat cells long before glucose intolerance--is insulin resistance initiated in the adipose tissue?. Int J Obes Relat Metab Disord.

[36] Cainzos-Achirica M, Fedeli U, Sattar N (2019). Epidemiology, risk factors, and opportunities for prevention of cardiovascular disease in individuals of South Asian ethnicity living in Europe. Atherosclerosis.

[37] Martin S, Sorokin EP, Thomas EL (2022). Estimating the effect of liver and pancreas volume and fat content on risk of diabetes: a mendelian randomization study. Diabetes Care.

[38] Yeung CHC, Au Yeung SL, Fong SSM, Schooling CM (2019). Lean mass, grip strength and risk of type 2 diabetes: a bi-directional Mendelian randomisation study. Diabetologia.

[39] Lear SA, Kohli S, Bondy GP, Tchernof A, Sniderman AD (2009). Ethnic variation in fat and lean body mass and the association with insulin resistance. J Clin Endocrinol Metab.

[40] Taylor R, Holman RR (2015). Normal weight individuals who develop type 2 diabetes: the personal fat threshold. Clin Sci (London, England : 1979).

