# Supplementary material for: Liver, visceral and subcutaneous fat in men and women of South Asian and white European descent: a systematic review and meta-analysis of new and published data
Source: Diabetologia. 2022 Oct 13;66(1):44–56. doi: 10.1007/s00125-022-05803-5 (PMC9729139; doi:10.1007/s00125-022-05803-5)
Supplement: Supplementary file 1 — (PDF 1350 kb) [file 125_2022_5803_MOESM1_ESM.pdf]

ESM Methods. Methods in studies by Ghouri et al and Iliodromiti et al

Data for liver and abdominal fat depots in studies by Ghouri et al and Iliodromiti et al undertaken at the University of Glasgow has not yet been published and this contains description of methods for data acquisition. Participants in study by Iliodromiti and colleagues were recruited as previously described and published [1]. MRI scans were performed by 3.0 Tesla (Magnetom, Siemens) MRI scanner at BHF Glasgow Cardiovascular research centre, Glasgow, UK. MRI scanner had dedicated transmit/receive coil positioned on participants anterior abdominal wall and plane localiser images were acquired by breath-hold axial T1-weighted images at level of L3-5. Data were acquired by two trained radiologists (Dr Stuart Ballantyne and Dr Jonathan Platt) and analysed with use of sliceOmatic software (version 4.3, TomoVision, Visual Imaging Inc., Canada). Each radiologist analysed scans independently and the inter-observer variation was estimated 2.5% for VAT and 0.4% for SAT. SliceOmatic software performs automatic segmentation of fat depots based on signal intensity cut-offs, but the user needs to review the automatic segmentation process to avoid any incorrect segmentations. The operator has control of sliding threshold but interface gives real time visual feedback on performance of threshold. Results are displayed in pixels and mm^2 for each compartment measured.

MRS liver fat data was obtained by 30×30×30mm MRS single voxel acquisition at the base of the liver (to minimise effects of respiratory motion) avoiding large vessels and at least one centimetre from the liver edge. Two consecutive spectra were acquired – with echo times (TE) of 144 ms and 35 ms (both with repetition times (TR) of 1500 ms) – in same location

for each participant using the point-resolved spectroscopy (PRESS) sequence. Manufacturers software (SAGE, GE healthcare, Milwaukee) was used to obtain liver fat and water ratios. Peak areas for resonances acquired and hepatic lipid resonances were quantified with reference to water resonance peak correcting for T_1 and T_2 [2]. Results could then be calculated for fat fraction (FF) measured as percentage using equation:

FA – area under fat peak, WA – area under water peak, CA- area under creatinine peak

$$FF\ (liver) = 100 \times FA / (FA + WA)$$

FF for the two different echo times every spectroscopy was different so a mean of the two readings was taken to represent fat percentage. This was done for each liver spectroscopy measurement respectively to calculate fat percentage.

Participants in study by Ghouri and colleagues were recruited as per study previously published and described [3]. MRS was performed for estimation of liver fat with a 1.5 Hdext GE Signa magnetic resonance scanner (GE Healthcare, Milwaukee) using an 8-channel cardiac coil at the Beatson West of Scotland cancer centre, Glasgow, UK. Scott Hanvey (SH), medical physicist, was responsible for performing the scans under the supervision of Dr John Foster (JF) consultant clinical scientist and deputy head for MR physics for NHS Greater Glasgow and Clyde. Consent was undertaken by

trained MRI staff working in the centre and clinical supervision and review of scans was provided by Dr Stuart Ballantyne, Consultant radiologist, Gartnavel General hospital. For analysis of abdominal SAT and VAT depots a T1-weighted single MRI slice was identified at level of L3-5 using plane localiser. Selected MRI image for each participant was then analysed for VAT and SAT volumes as described above using sliceOmatic software (version 4.3, TomoVision, Visual Imaging Inc., Canada). Dr James McLaren (JM) and Dr Stamatina Iliodromiti (SI) analysed images independently using sliceOmatic software and mean of two results was taken as measured value for data by Ghouri and colleagues.

[1] Iliodromiti S, Ghouri N, Celis-Morales CA, Sattar N, Lumsden MA, Gill JM (2016) Should Physical Ativity Recommendations for South Asian Adults Be Ethnicity-Specific? Evidence from a Cross-Sectional Study of South Asian and White European Men and Women. PloS one 11(8): e0160024. 10.1371/journal.pone.0160024
[2] Naressi A, Couturier C, Devos JM, et al. (2001) Java-based graphical user interface for the MRUI quantitation package. MAGMA 12(2-3): 141-152
[3] Ghouri N, Purves D, McConnachie A, Wilson J, Gill JM, Sattar N (2013) Lower cardiorespiratory fitness contributes to increased insulin resistance and fasting glycaemia in middle-aged South Asian compared with European men living in the UK. Diabetologia 56(10): 2238-2249. 10.1007/s00125-013-2969-y

ESM Table 1. Demographic and cardiometabolic risk factor profile of South Asians and White Europeans stratified by sex in the unpublished studies by Iliodromiti et al and Ghouri et al.

	South Asian women	White European women	South Asian men	White European men
N	44	55	28	24
Age (years)	53.5 (10.3)	54.8 (7.0)	49.8 (8.2)	50.3 (5.9)
BMI ($kg.m^{-2}$)	27.0 (5.3)	27.1 (4.5)	28.5 (4.8)	27.4 (3.2)
Waist (cm)	83.3 (12.1)	81.8 (9.9)	101.1 (12.5)	96.8 (7.3)
Non Smoker (n, %)	45 (98%)	49 (89%)	26 (93%)	23 (96)
Menopause (n, %)	28 (61%)	33 (60%)	NA	NA
Glucose ($mmol.l^{-1}$)	5.00 [4.60, 5.30]	5.00 [4.70, 5.20]	5.25 [4.90, 5.70]	5.00 [4.80, 5.55]
HbA1c (mmol/mol)	39.0 [36.0, 41.0]	34.0 [31.0, 37.0]	37.7 [36.6, 40.2]	35.5 [34.2, 37.7]
HbA1c (%)[a]	5.72 [5.44, 5.90]	5.26 [4.99, 5.54]	5.60 [5.50, 5.83]	5.40 [5.28, 5.60]
Insulin ($pmol.l^{-1}$)	60 [41, 88]	44 [31, 58]	91 [58, 118]	61 [40, 74]
$HOMA_{IR}$[a]	2.22 [1.50, 3.28]	1.64 [1.07, 2.18]	3.36 [2.41, 4.44]	2.27 [1.39, 2.99]
Total Cholesterol ($mmol.l^{-1}$)	5.39 (0.82)	5.62 (0.74)	5.33 (0.83)	5.39 (0.98)
HDL-Cholesterol ($mmol.l^{-1}$)	1.46 (0.36)	1.73 (0.48)	1.11 (0.17)	1.27 (0.24)
LDL-cholesterol ($mmol.l^{-1}$)	3.29 (0.68)	3.35 (0.83)	3.49 (0.71)	3.44 (0.84)
Triacylglycerol ($mmol.l^{-1}$)	1.28 [0.87, 1.72]	0.90 [0.70, 1.27]	1.35 [0.98, 2.00]	1.20 [0.93, 1.68]
Systolic blood pressure (mmHg)	127.4 (14.2)	127.0 (18.3)	123.9 (11.9)	129.3 (12.4)
Diastolic blood pressure (mmHg)	79.2 (10.7)	78.6 (10.3)	76.5 (7.0)	76.8 (6.9)
Subcutaneous fat (cm^2)	243.4 [197.8, 323.7]	262.4 [190.2, 319.8]	177.5 (63.6)	122.7 (38.8)
Visceral fat (cm^2)	109.4 [76.2, 165.6]	81.5 [56.7, 120.6]	186.1 (58.3)	213.9 (63.5)
Liver fat (%)	4.3 [2.0, 9.2]	2.5 [1.1, 5.9]	6.92 (2.11)	7.52 (2.35)

Values are mean (SD), median [IQR], or n (%).

ESM Table 2. Demographic summary data (summary mean and range) for the studies included in the meta-analyses on subcutaneous and visceral fat.

	South Asian men	European men	South Asian non-diabetic men	European non-diabetic men	South Asian women	European women	South Asian non-diabetic women	European non-diabetic women
N	1156	2891	615	2274	697	2271	402	1402
Age (years)	48.2 (22 to 69)	49.3 (22 to 70)	42 (22 to 58)	42.3 (22 to 62)	51.6 (24 to 68)	53.4 (25 to 70)	46.5 (24 to 58)	47.6 (25 to 62)
BMI (kg.m^{-2})	25.6 (21 to 28)	26.3 (22 to 28)	25.1 (21 to 27)	25.5 (22 to 27)	26.3 (22 to 29)	27.2 (23 to 29)	25.2 (22 to 27)	26.3 (23 to 29)
Glucose (mmol.l^{-1})	5.3 (5.1 to 5.4)	5.1 (5.0 to 5.3)	5.3 (5.1 to 5.4)	5.1 (5.0 to 5.3)	5.0 (4.8 to 5.2)	4.9 (4.8 to 5)	5.0 (4.8 to 5.2)	4.9 (4.8 to 5)
Insulin (pmol.l^{-1})	59 (43 to 91)	51 (29 to 75)	62 (42 to 90)	45 (30 to 63)	56 (46 to 66)	53 (44 to 72)	55 (46 to 66)	53 (44 to 72)

n = 6 and 5 out of 9 studies had summary data on glucose and insulin levels respectively for men and 5 out of 8 for women

ESM Table 3. Demographic summary data (summary mean and range) for the studies included in the meta-analyses on liver fat.

	South Asian men	European men	South Asian non-diabetic men	European non-diabetic men	South Asian women	European women	South Asian non-diabetic women	European non-diabetic women
N	677	2394	611	2328	575	2076	509	2001
Age (years)	37.9 (22 to 58)	38 (22 to 58)	36.7 (22 to 58)	36.2 (22 to 58)	50.8 (38 to 58)	52.7 (35 to 62)	52.1 (38 to 58)	53.2 (35 to 62)
BMI ($kg.m^{-2}$)	25.3 (21 to 28)	25.8 (22 to 28)	24.9 (21 to 27)	25.4 (22 to 28)	26.4 (26 to 28)	27.5 (27 to 29)	26 (26 to 27)	27.1 (27 to 29)
Glucose ($mmol.l^{-1}$)	5.3 (5.1 to 5.4)	5.1 (5.0 to 5.3)	5.3 (5.1 to 5.4)	5.1 (5.0 to 5.3)	5.1 (4.9 to 5.2)	4.9 (4.8 to 5)	5.1 (4.9 to 5.2)	4.9 (4.8 to 5)
Insulin ($pmol.l^{-1}$)	63 (43 to 91)	51 (29 to 75)	66 (42 to 90)	46 (30 to 63)	54 (46 to 62)	48 (44 to 53)	52 (46 to 60)	47 (44 to 49)

n = 6 out of 7 studies had summary data on glucose and insulin levels for men and 4 out of 5 for women

ESM Table 4. Study-level risk of bias for studies of VAT/SAT using a preliminary version of the ROBINS-E tool

Studies	Confounding	Participant selection	Measurement of exposure	Departures from exposures	Missing data	Measurement of outcomes	Selection of reported results	Overall study-level bias
Anand et al [12]	Moderate[2]	Low	Low	Low	Low	Moderate[4]	Low	Moderate
Bakker et al [32]	Low	Low	Low	Low	Low	Moderate[4]	Low	Moderate
Chandalia et al [13]	Low	Low	Low	Low	Low	Moderate[4]	Low	Moderate
Eastwood et al [21]	Serious[1,3]	Low	Low	Low	Low	Moderate[4]	Low	Serious
Lear et al [22-23]	Moderate[2]	Low	Low	Low	Low	Moderate[4]	Low	Moderate
Shah et al [28]	Moderate[1,2]	Low	Low	Low	Low	Moderate[4,5]	Low	Moderate
Szuszkiewicz-Garcia et al [31]	Moderate[2]	Low	Low	Low	Low	Moderate[4]	Low	Moderate
UK Biobank	Low	Low	Low	Low	Low	Low	Low	Low
Ghouri et al	Low	Low	Low	Low	Low	Moderate[4]	Low	Moderate
Iliodromiti et al	Low	Low	Low	Low	Low	Moderate[4]	Low	Moderate

[1]men not matched for BMI, [2]women not matched for BMI, [3]participants with diabetes included, [4]non-blinded measurement of outcome or unclear whether measurement was blinded, [5]different MRI scanner used at different study sites

ESM Table 5. Study-level risk of bias for studies of liver fat using a preliminary version of the ROBINS-E tool

Studies	Confounding	Participant selection	Measurement of exposure	Departures from exposures	Missing data	Measurement of outcomes	Selection of reported results	Overall study-level bias
Anand et al [12]	Moderate[2]	Low	Low	Low	Low	Moderate[3]	Low	Moderate
Dick et al [24]	Low	Low	Low	Low	Low	Moderate[3]	Low	Moderate
Petersen et al [25]	Low	Low	Low	Low	Low	Moderate[3]	Low	Moderate
Shah et al [28]	Moderate[1,2]	Low	Low	Low	Low	Moderate[3,4]	Low	Moderate
UK Biobank	Low	Low	Low	Low	Low	Low	Low	Low
Ghouri et al	Low	Low	Low	Low	Low	Moderate[3]	Low	Moderate
Iliodromiti et al	Low	Low	Low	Low	Low	Moderate[3]	Low	Moderate

[1]men not matched for BMI, [2]women not matched for BMI, [3]non-blinded measurement of outcome or unclear whether measurement was blinded, [4]different MRI scanner used at different study sites

ESM Table 6. GRADE certainty of evidence for studies included in meta-analysis

		Quality assessment					Overall Certainty
	Number of studies (No of participants)	Study limitations[1]	Consistency	Directness	Precision[2]	Publication bias	
Abdominal fat male studies	9 (4047)	Serious limitations	Inconsistent results with high heterogeneity	Direct	No important imprecision	Possible bias due to asymmetry on funnel plots for subcutaneous fat (not visceral fat)	Low
Abdominal fat female studies	8 (2968)	Serious limitations	Inconsistent results with high heterogeneity	Direct	No important imprecision	Possible bias due to asymmetry of funnel plots	Low
Liver fat male studies	7 (3071)	Serious limitations	Inconsistent results with high heterogeneity	Direct	No important imprecision	Unlikely due to symmetry of funnel plots	Low
Liver fat female studies	5 (2651)	Serious limitations	Moderate heterogeneity	Direct	No important imprecision	Unlikely due to symmetry of funnel plots	Low

[1]study limitations include differences in BMI between groups in some studies, including of participants with diabetes in one study, and non-blinding of outcome assessors in all studies. In sensitivity analyses excluding studies which included patients with diabetes, and only including studies where BMI was matched, the overall findings were unchanged.

[2] large number of participants with consistent results and narrow CI in random effects analysis

A

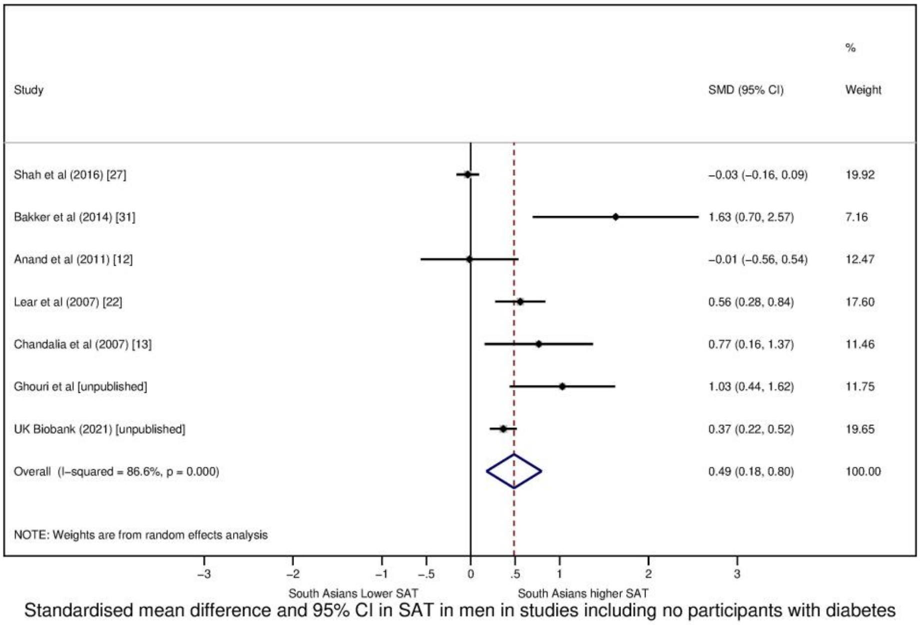

B

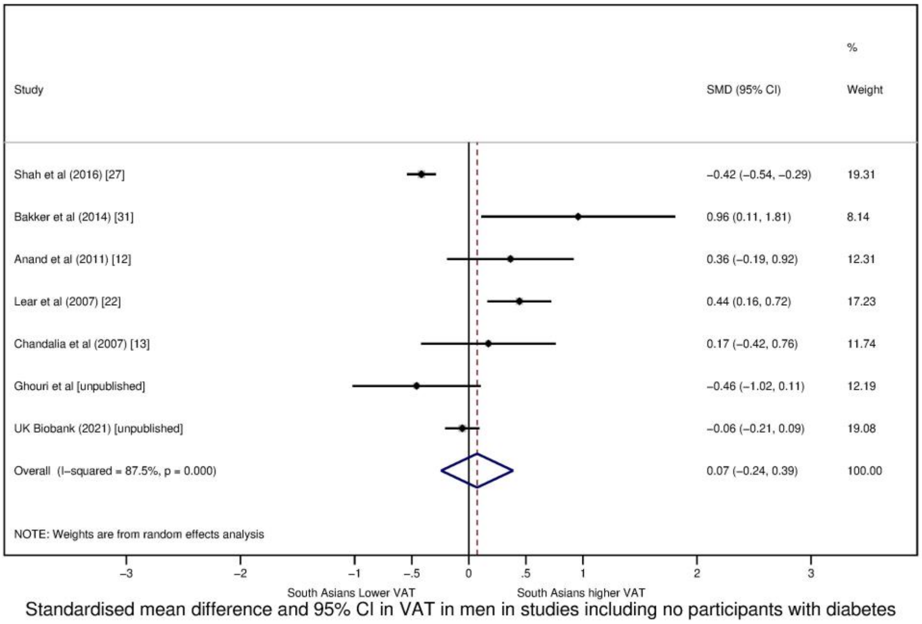

ESM Fig 1. Standardised mean differences in subcutaneous (SAT, A), and visceral (VAT, B) in South Asian versus white European men in studies including no participants with diabetes.

A

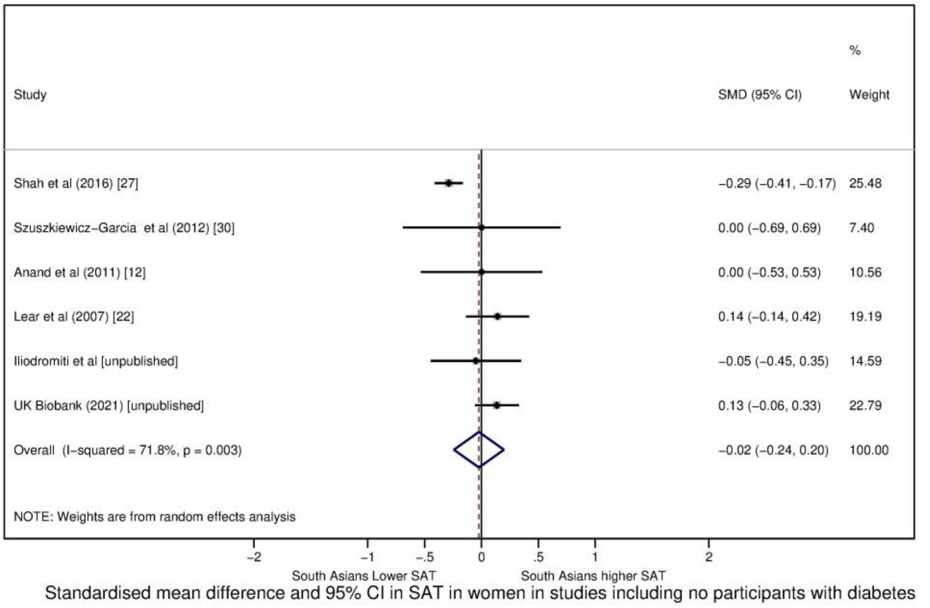

B

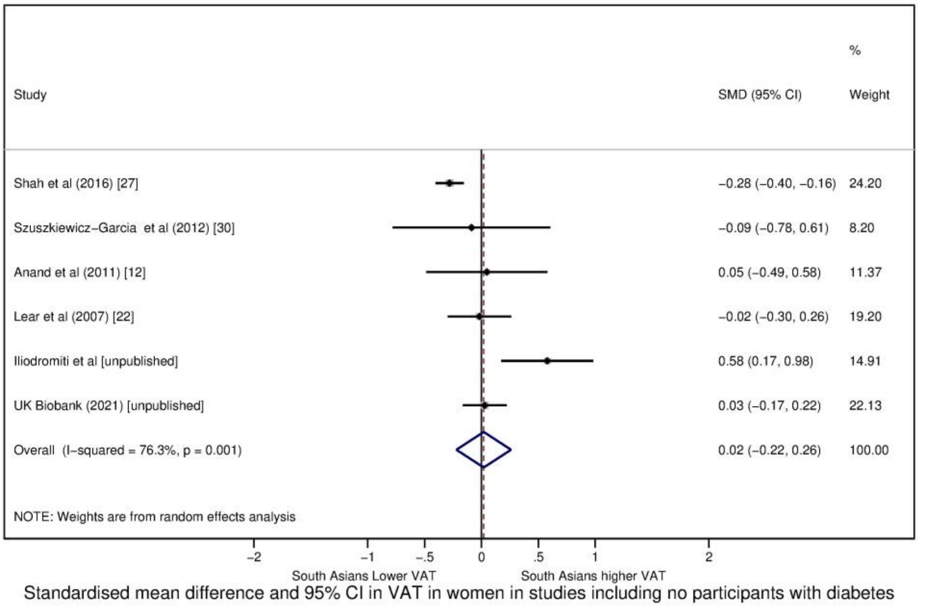

ESM Fig 2. Standardised mean differences in subcutaneous (SAT, A), and visceral (VAT, B) in South Asian versus white European women in studies including no participants with diabetes.

A

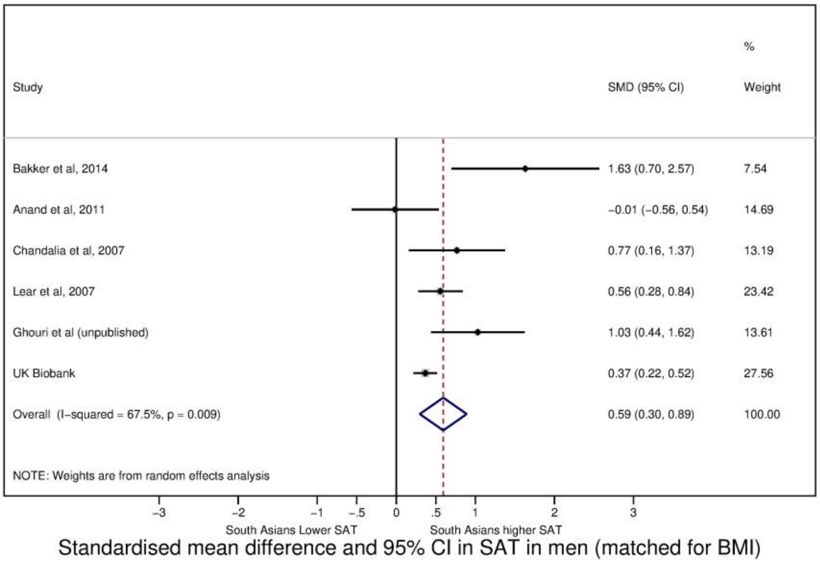

B

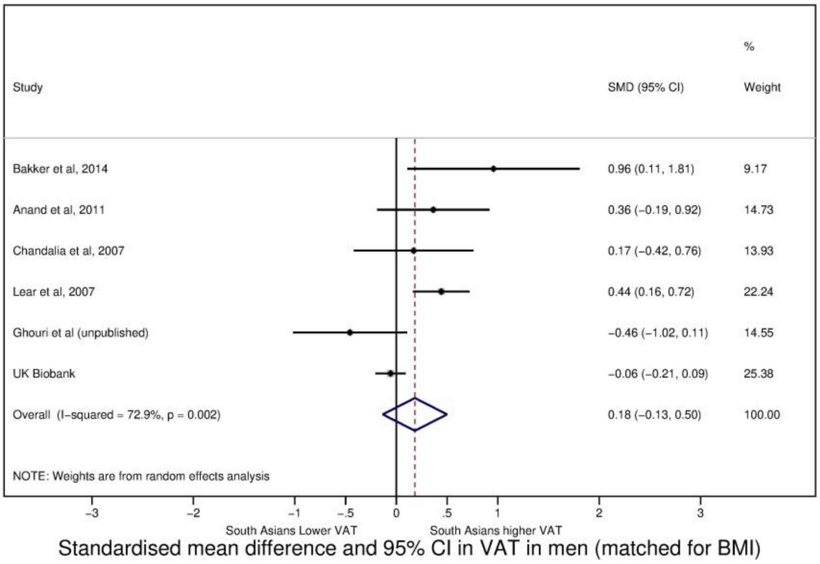

C

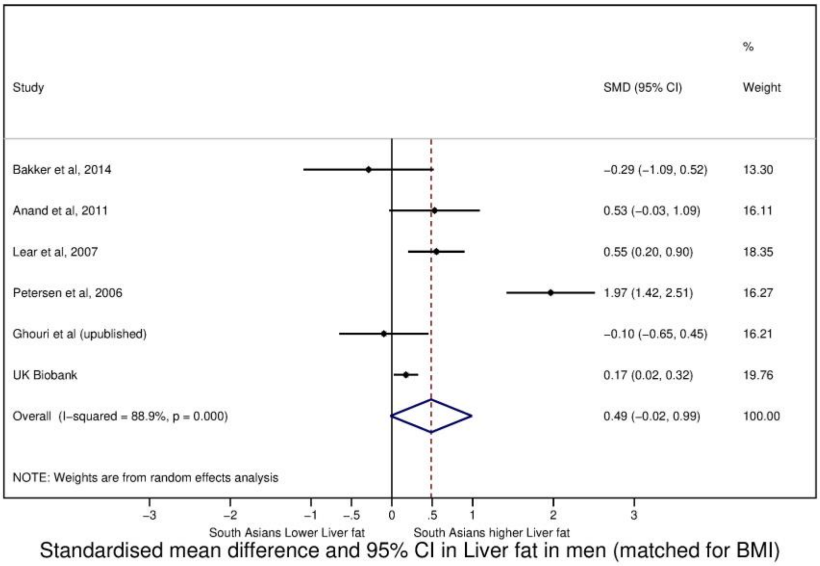

ESM Fig 3. Standardised mean differences in subcutaneous (SAT, A), visceral (VAT, B) and liver fat (C) in South Asian versus White European men in studies with matched BMI.

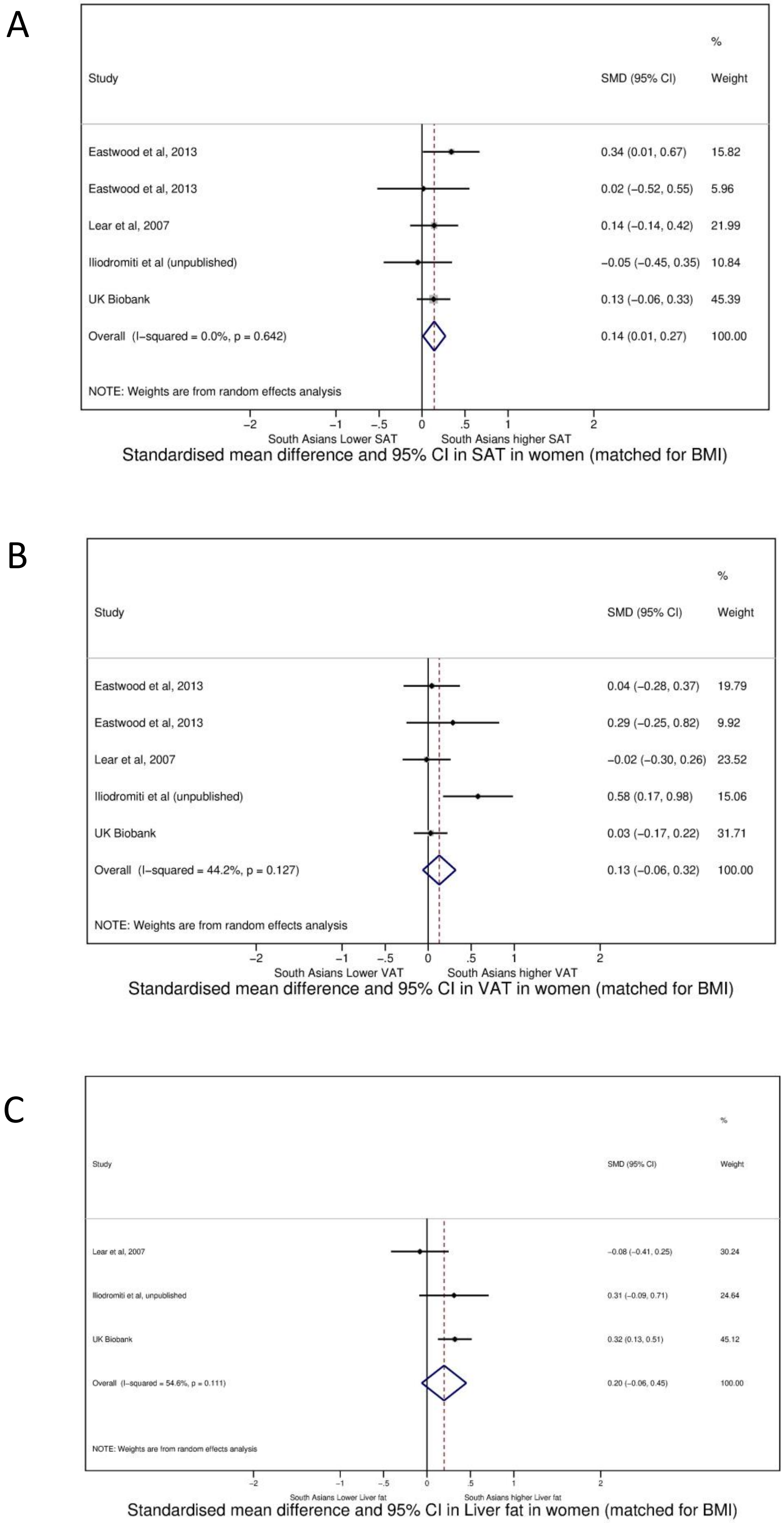

ESM Fig 4. Standardised mean differences in subcutaneous (SAT, A), visceral (VAT, B) and liver fat (C) in South Asian versus White European women in studies with matched BMI.

A

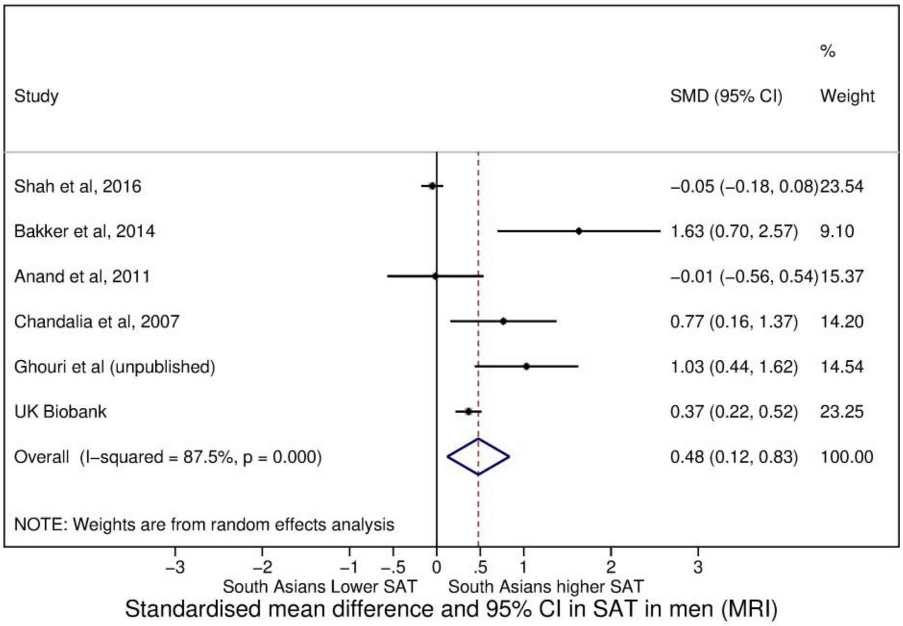

B

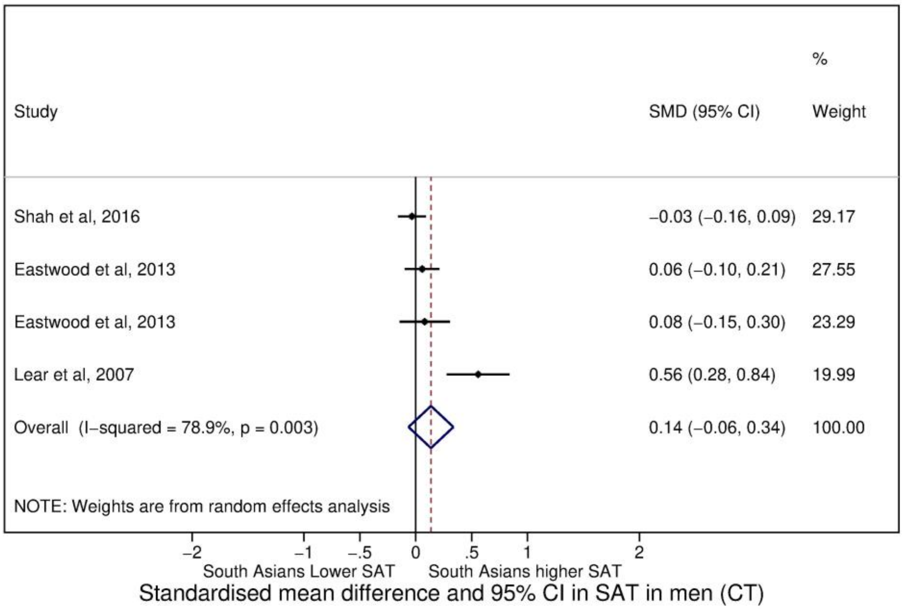

ESM Fig 5. Standardised mean differences in subcutaneous fat (SAT) in South Asian versus White European men in studies using MRI (A) and CT (B) as the assessment tool.

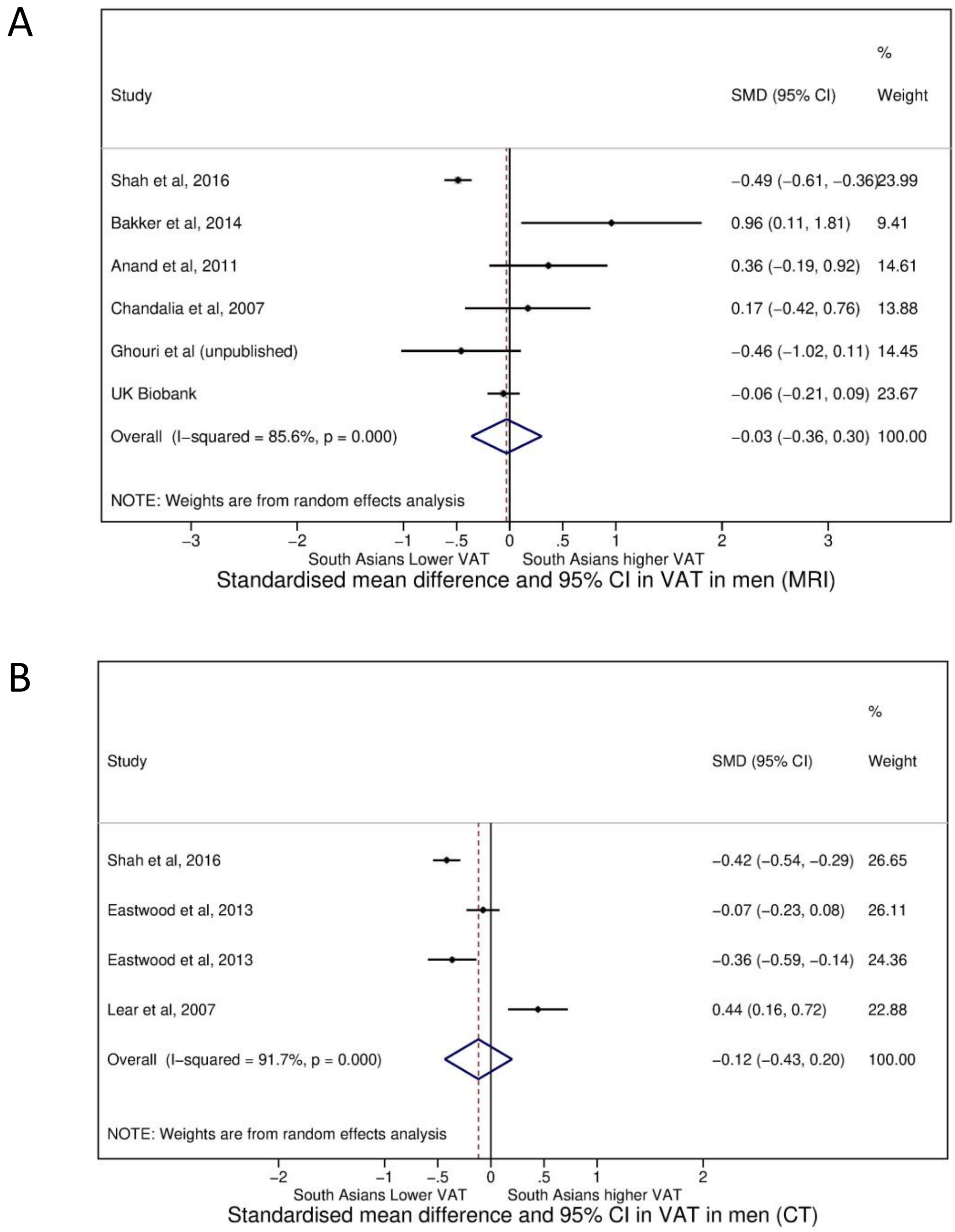

ESM Fig 6. Standardised mean differences in visceral (VAT) in South Asian versus White European men in studies using MRI (A) and CT (B) as the assessment tool.

A

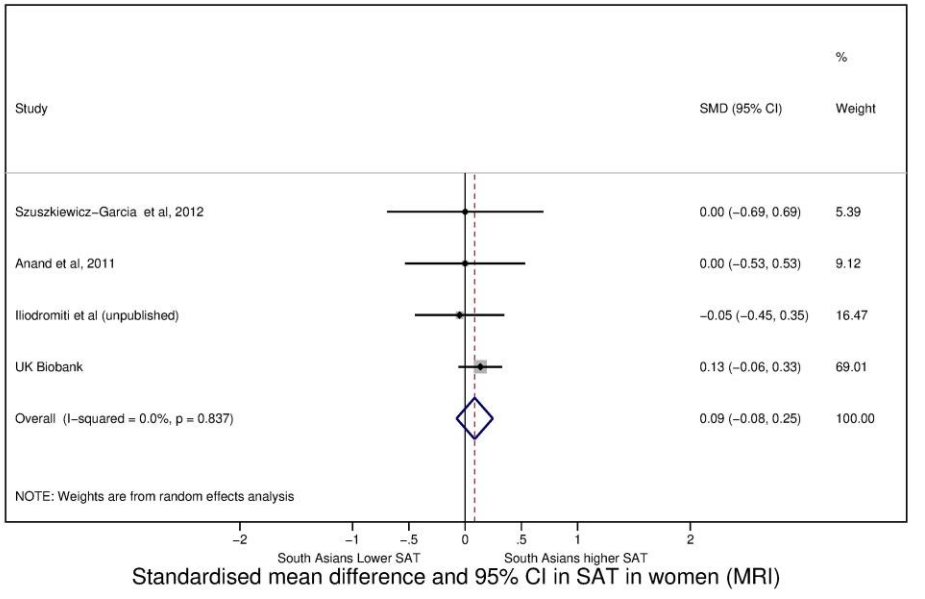

B

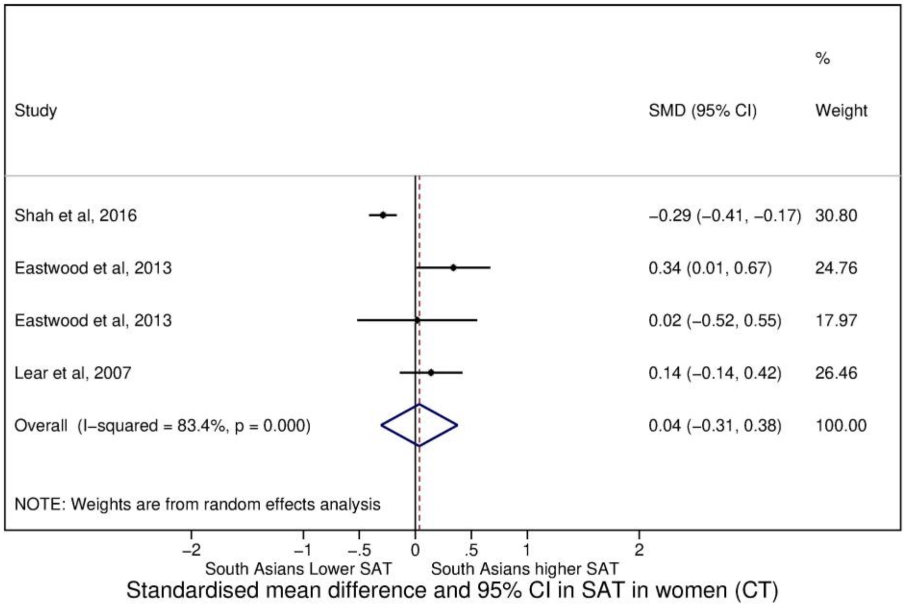

ESM Fig 7. Standardised mean differences in subcutaneous fat (SAT) in South Asian versus White European women in studies using MRI (A) and CT (B) as the assessment tool.

A

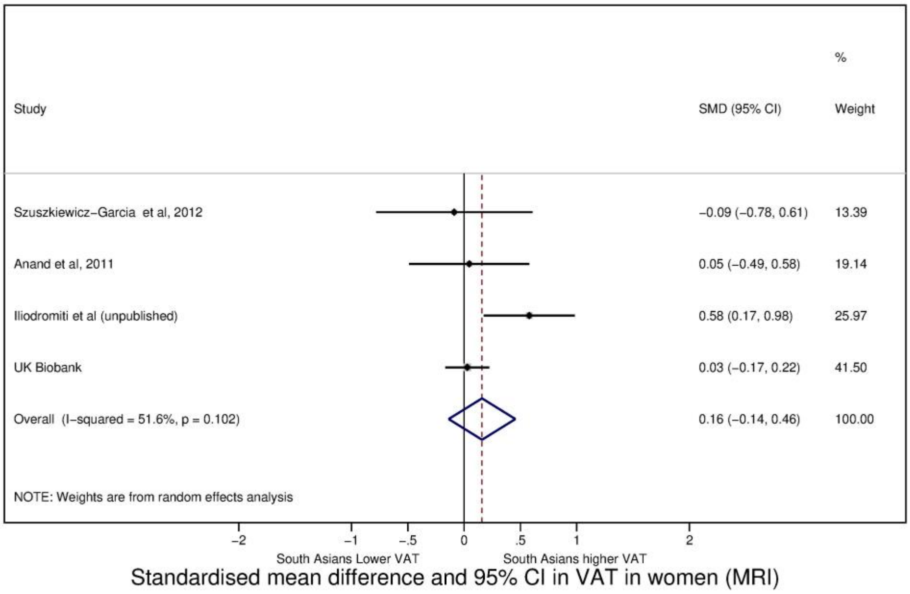

B

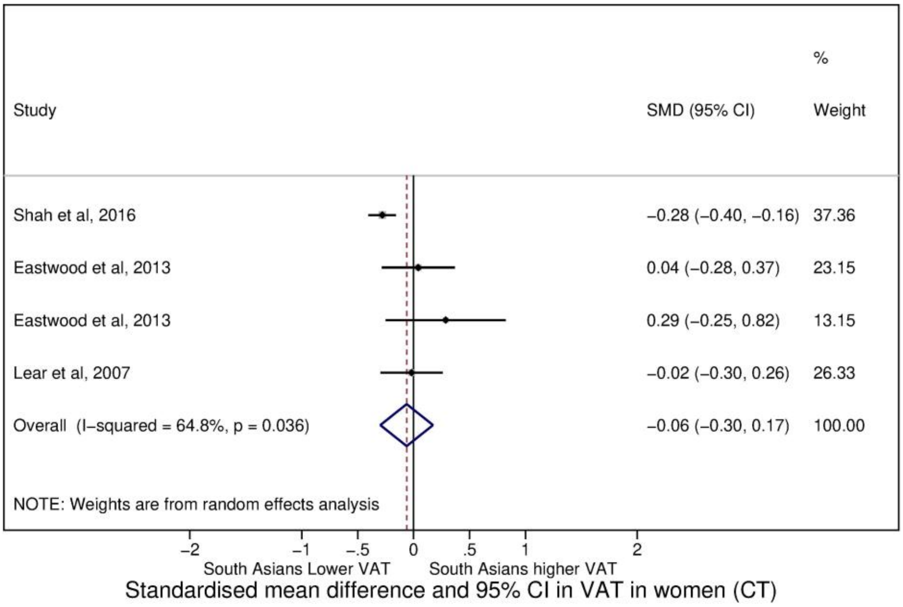

ESM Fig 8. Standardised mean differences in visceral (VAT) in South Asian versus White European women in studies using MRI (A) and CT (B) as the assessment tool.

A: SAT men

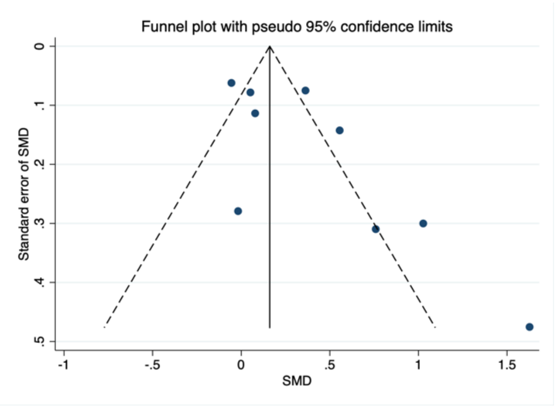

B: SAT women

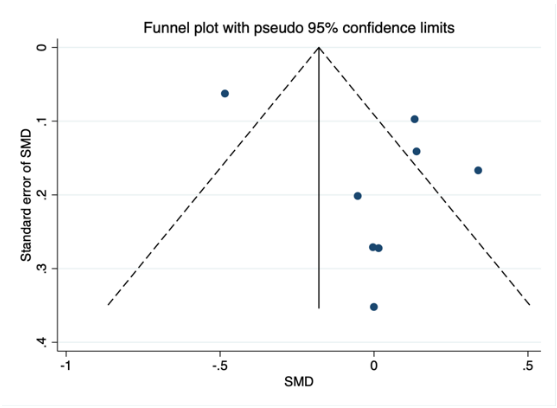

C: VAT men

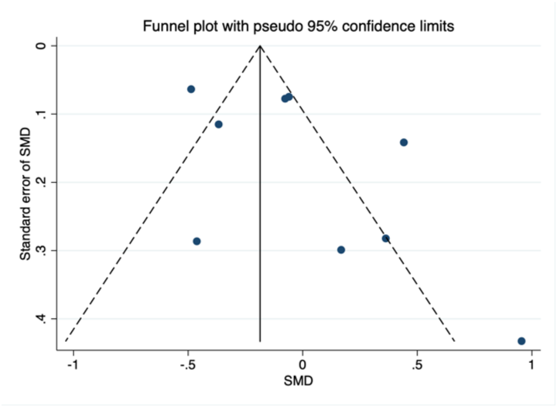

D: VAT women

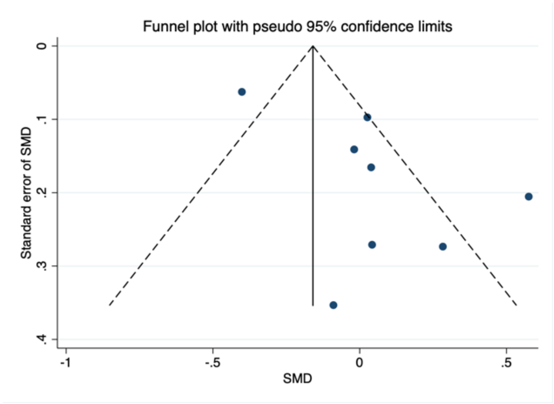

E: Liver fat men

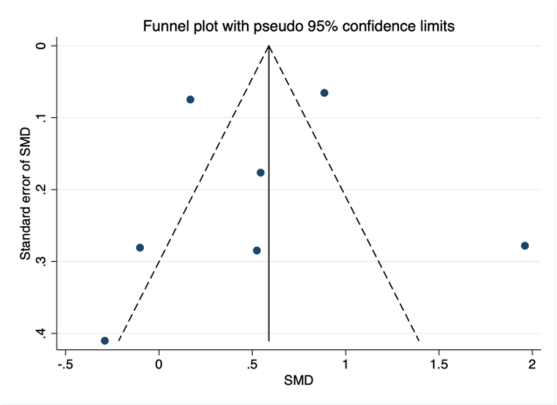

F: Liver fat women

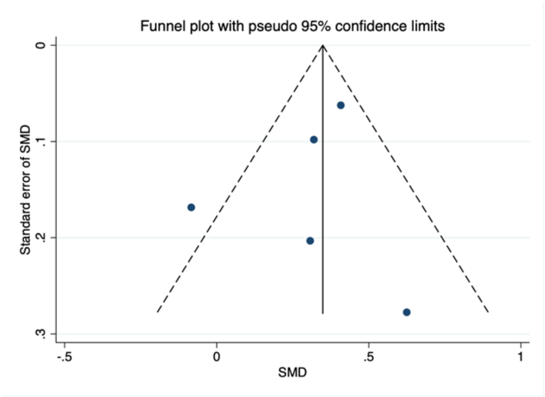

ESM Fig 9. Funnel plots assessing the risk of publication bias for comparison of subcutaneous (SAT; men A, women B), visceral (VAT; men C, women D) and liver (men E, women F) fat in studies of men and women.
